# Transcriptomics reveals specific molecular mechanisms underlying transgenerational immunity in *Manduca sexta*

**DOI:** 10.1002/ece3.6764

**Published:** 2020-09-17

**Authors:** Charles L. Roesel, Rebeca B. Rosengaus, Wendy Smith, Steven V. Vollmer

**Affiliations:** ^1^ Marine Science Center Northeastern University Nahant MA USA

**Keywords:** insects, invertebrates, life history evolution, transcriptomics, transgenerational immune priming

## Abstract

The traditional view of innate immunity in insects is that every exposure to a pathogen triggers an identical and appropriate immune response and that prior exposures to pathogens do not confer any protective (i.e., adaptive) effect against subsequent exposure to the same pathogen. This view has been challenged by experiments demonstrating that encounters with sublethal doses of a pathogen can prime the insect's immune system and, thus, have protective effects against future lethal doses. Immune priming has been reported across several insect species, including the red flour beetle, the honeycomb moth, the bumblebee, and the European honeybee, among others. Immune priming can also be transgenerational where the parent's pathogenic history influences the immune response of its offspring. Phenotypic evidence of transgenerational immune priming (TGIP) exists in the tobacco moth *Manduca sexta* where first‐instar progeny of mothers injected with the bacterium *Serratia marcescens* exhibited a significant increase of in vivo bacterial clearance. To identify the gene expression changes underlying TGIP in *M. sexta*, we performed transcriptome‐wide, transgenerational differential gene expression analysis on mothers and their offspring after mothers were exposed to *S. marcescens*. We are the first to perform transcriptome‐wide analysis of the gene expression changes associated with TGIP in this ecologically relevant model organism. We show that maternal exposure to both heat‐killed and live *S. marcescens* has strong and significant transgenerational impacts on gene expression patterns in their offspring, including upregulation of peptidoglycan recognition protein, toll‐like receptor 9, and the antimicrobial peptide cecropin.

## Graphical abstract

To identify the gene expression changes underlying TGIP in *Manduca sexta*, we performed transcriptome‐wide, transgenerational differential gene expression analysis on mothers and their offspring after mothers were exposed to *S. marcescens*. We show that maternal exposure to both heat‐killed and live *S. marcescens* has strong and significant transgenerational impacts on gene expression patterns in their offspring, including upregulation of peptidoglycan recognition protein, toll‐like receptor 9, and the antimicrobial peptide cecropin.

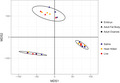

## 1 INTRODUCTION

The phenomenon of transgenerational immune priming (TGIP) has received significant attention, especially in insect systems. Organisms exposed to stressful environmental selection pressures can increase the survival of their offspring by priming their progeny with beneficial nutrients, hormones, and epigenetic factors (Knorr, Schmidtberg, Arslan, Bingsohn, & Vilcinskas, 2015; Pigeault, Garnier, Rivero, & Gandon, 2016; Trauer‐Kizilelma & Hilker, 2015a, 2015b). Insects can also respond to pathogenic pressures by priming their progeny's immune systems. TGIP has been demonstrated in a variety of invertebrate taxa (Barribeau, Schmid‐Hempel, & Sadd, 2016; Hanif, Bakopoulos, & Dimitriadis, 2004; Knorr et al., 2015; Pigeault et al., 2016; Roth, Beemelmanns, Barribeau, & Sadd, 2018; Sadd, Kleinlogel, Schmid‐Hempel, & Schmid‐Hempel, 2005; Salmela, Amdam, & Freitak, 2015; Trauer‐Kizilelma & Hilker, 2015a), but the mechanisms of inheritance following immune challenge are often not well characterized (Hernández López, Schuehly, Crailsheim, & Riessberger‐Gallé, 2014; Mcnamara, Lieshout, & Simmons, 2014; Norouzitallab, Baruah, Biswas, Vanrompay, & Bossier, 2016; Roth et al., 2018; Trauer‐Kizilelma & Hilker, 2015a).

One mechanism of transgenerational immunity which has been experimentally validated in insects involves the translocation of bacteria (Freitak et al., 2014) or bacterial cell wall components from mothers to their offspring (Salmela et al., 2015). In the honeycomb moth *Galleria mellonella*, Freitak et al. (2014) used fluorescently labeled, heat‐killed *Escherichia coli* to trace the transfer of bacteria from the guts of mothers through the hemocoel and ovarioles into their developing eggs. They reported upregulation of prophenoloxidase, peptidoglycan recognition protein, glutathione‐s‐transferase, and lipopolysaccharide‐binding protein in eggs of immune‐challenged mothers. Salmela et al. (2015) identified vitellogenin protein as the carrier required for the transport of *E. coli* cell wall fragments into the developing eggs of the European honeybee *Apis mellifera*. In addition, several studies provide evidence of epigenetic transmission. Eggert, Kurtz, and Diddens‐de Buhr (2014) reported that paternal transgenerational immunity in the beetle *Tribolium castaneum* exposed to *Bacillus thuringiensis* is passed via sperm, and to a lesser degree seminal fluid, suggesting that epigenetic modifications were involved.

The *Manduca* immune response to pathogens begins when pattern recognition receptors (PRRs) like hemolin, peptidoglycan recognition proteins, β‐1,3‐glucan recognition proteins, toll‐like receptors (TLRs), and C‐type lectins bind to microbial surface molecules (Yu, Zhu, Ma, Fabrick, & Kanost, 2002), triggering phagocytosis, nodule formation, encapsulation, melanization, and synthesis of antimicrobial peptides/proteins (Horohov & Dunn, 1983). Antimicrobial proteins identified in *M. sexta* hemolymph include lysozyme, cecropins, and attacin (Kanost, Jiang, & Yu, 2004). The fat body, analogous to vertebrate adipose tissues and liver, is the major source of insect plasma proteins involved in the insect immune response (He et al., 2016). In response to gram‐positive *Micrococcus lysodeikticus*, fat bodies of *Manduca* upregulate proteins such as prophenoloxidase‐activating proteinase, mannan‐binding lectin serine proteinase, scolexin A, urokinase‐type plasminogen activator, peptidoglycan recognition protein, immulectin, lipopolysaccharide‐binding protein, lebocin, and gloverin (Zhu, Johnson, Myers, & Kanost, 2003). A similar upregulation of fat body proteins has been documented in response to gram‐negative *Photorhabdus* spp. include hemolin, immulectin‐2, and peptidoglycan recognition protein (Eleftherianos, Millichap, Ffrench‐Constant, & Reynolds, 2006). Ao, Ling, and Yu (2008) also reported an upregulation of toll‐like receptor in *Manduca* exposed to gram‐negative *E. coli*.

Evidence for transgenerational immunity in *Manduca sexta* has also been recently documented in the offspring of mothers exposed to heat‐killed and live *Serratia marcescens* bacteria (Rosengaus et al., 2017). Rosengaus et al. (2017) demonstrated that maternal pathogen exposure significantly affected in vivo bacterial clearance by their offspring. Using an in vivo “clearance of infection” assay, they showed that first‐instar larvae, offspring of *Manduca* females injected with either live or heat‐killed *S. marcescens* had significantly lower microbial loads 24 and 48 hr after injection of live *Serratia* than offspring of females injected with a sterile saline control. Here, we used transcriptome‐wide RNA sequencing to identify gene expression changes underlying the transgenerational phenotypic effects reported by Rosengaus et al. (2017). We compared gene expression patterns in fat body and ovariole tissues of mothers exposed to live and heat‐killed *S. marcescens* and then compared gene expression patterns in their embryos to identify any transgenerational impacts on gene expression due to maternal pathogen exposure.

The gram‐negative bacterium, *S. marcescens*, was chosen to elicit a maternal immune response because it is an ecologically relevant pathogen, commonly found on foliage and in soil (Sikorowski, Lawrence, & Inglis, 2001). *Serratia marcescens* is likely also encountered by developing larvae during herbivory and during the fifth and final larval stage when larvae wander and/or when they pupate subterraneously. In addition, our prior experiments on pathogen‐induced maternal effects, which resulted in in vivo evidence of enhanced immune responsiveness across generations, were also carried out with the same strain of *Serratia* as this work (Rosengaus et al., 2017). It was important for us to maintain consistency with regard to the pathogenic strain so that we could transpose the current molecular data onto the in vivo clearing assay results of 2017.

Although previous work on *Manduca* included transcriptomic analyses of specific genes (Lee & Horodyski, 2006) in the context of starvation and mating, transcriptome‐wide analyses for sex chromosome differences (Smith, Chen, Blissard, & Briscoe, 2014) and within‐generation immune challenges (Van Munster et al., 2007), and phenotypic analyses of TGIP (Rosengaus et al., 2017) the present work, based on genome‐wide transcriptomic analyses, provide more nuanced information with respect to gene expression changes involved in TGIP.

## 2 METHODS

*Manduca sexta* eggs were obtained from Carolina Biological Supply (Burlington, NC), then reared, and treated following the same protocol used by Rosengaus et al. (2017). Briefly summarizing, larvae were reared on a standard artificial diet (Bell & Joachim, 1976) at 25°C under a 16‐hr:8‐hr light/dark cycle. Two days prior to expected eclosion, female pupae were swabbed with 70% ethanol and injected with sterile saline (10 μl, *n* = 8), heat‐killed *Serratia* (10 μl at 1 × 10^8^ cells/ml, *n* = 8), or live *Serratia* (10 μl at 4 × 10^5^ CFU/ml, *n* = 8) using a 10 μl Hamilton syringe and sterile needle. After eclosion, twelve of the treated females (4 each from saline, heat‐killed, and live exposures) were sacrificed and RNA was extracted from ovarioles and fat bodies to profile their transcriptomic response to pathogen exposure. Total RNA was extracted from fat bodies and ovarioles of each adult female (*n* = 4 from the saline‐treated mothers, *n* = 4 from the heat‐killed mothers, and *n* = 4 from the live *S. marcescens*‐injected mothers) using the Promega SV Total RNA Isolation Kit.

The twelve remaining females (4 each from saline, heat‐killed, and live) were mated with untreated (naïve) males following the monogamous mating protocol: A single treated female paired with an untreated male inside a cage (30 × 30 × 60 cm) (Rosengaus et al. (2017). The mated moths oviposited on 30 mm tobacco extract‐infused foam plugs suspended from the cage ceiling. Twenty‐four hours post‐oviposition, embryos were collected and sacrificed for RNA extraction to profile the transgenerational transcriptomic response to maternal pathogen exposure. Total RNA was extracted from whole embryos (*n* = 12) using the Promega SV Total RNA Isolation Kit.

Three samples for each tissue/treatment group were selected for library preparation for a total of 27 RNA samples. mRNA was isolated using the NEBNext^®^ Poly(A) mRNA Magnetic Isolation Module, Illumina libraries were produced using the NEBNext^®^ Ultra™ Directional RNA Library Prep Kit for Illumina^®^, and the libraries were sequenced at the Bauer Core Facility of Harvard University. The ovariole and fat body libraries were sequenced on two lanes in rapid‐run paired‐100 mode on an Illumina HiSeq 2500. The sequencing run produced a total of 45,260,841 read pairs for fat body and 130,646,641 read pairs for ovariole. The embryo libraries were sequenced on one lane in rapid‐run paired‐250 mode producing a total of 38,189,468 reads. The reads were adapter‐ and quality‐trimmed using Trimmomatic version 0.36 (Bolger, Lohse, & Usadel, 2014) using a 4‐base sliding‐window quality cutoff of 30 (Phred + 33) and the TruSeq3 adapter sequence file (TruSeq3‐PE.fa). Transcript counts were quantified against *M. sexta* predicted coding sequences published by Kanost et al. (2016) using Salmon (Patro, Duggal, Love, Irizarry, & Kingsford, 2017). Transcript counts were imported into DESeq2 (Love, Huber, & Anders, 2014) using tximport (Soneson, Love, & Robinson, 2015).

### 2.1 Gene expression analysis

MDS and PERMANOVA analyses were used to identify transcriptome‐wide differences in gene expression due to immune treatment (saline, heat‐killed *Serratia*, live *Serratia*) and tissue type (adult fat body, adult ovariole, whole embryo). Hellinger‐transformed, DESeq2‐normalized (Love et al., 2014) counts for each tissue type were analyzed using PERMANOVA to identify transcriptome‐wide differences in expression patterns using the adonis function within the R package Vegan (Oksanen, Blanchet, & Friendly, 2019) with 10,000 permutations.

A two‐factor negative binomial GLM implemented in DESeq2 (Love et al., 2014) was used to compare saline versus heat‐killed and live *S. marcescens* exposures in the two maternal tissues (ovarioles and fat bodies) and their embryos to identify TGIP. This approach allows us to identify differentially expressed (DE) genes and then characterize the function of those genes using protein annotations and KEGG pathway analyses.

### 2.2 Annotation

To facilitate KEGG pathway analysis, differentially expressed transcripts were mapped to KEGG ortholog IDs. The transcripts were aligned to Swiss‐Prot (The Uniprot Consortium, 2017) using blastx (Camacho et al., 2009). Swiss‐Prot hits were filtered using an e‐value cutoff of 1e−5 and matched to KEGG orthologs using the KEGG API. For each *Manduca* transcript, the KEGG ortholog corresponding to the lowest BLAST e‐value was selected.

## 3 RESULTS

RNA‐Seq data were generated from 27 samples, three ereplicates for each tissue type (adult fat body, adult ovariole, and embryos) and treatment group (saline, heat‐killed, and live *S. marcescens*) combination. The number of mapped RNA‐seq reads per sample averaged 5,407,438 (±1,528,155 SE). The Friedman rank tests showed no significant mapping differences due to tissue type (*X* ^2^ = 0.667, *df* = 2, *p*‐value = .717 or treatment (*X* ^2^ = 0.667, *df* = 2, *p*‐value = .717). The means of mapped reads by tissue type were 3,371,322 (± 456,357 SE) for fat body, 10,563,659 (± 4,180,785 SE) for ovariole, and 2,287,335 (± 152,030 SE) for embryo.

### 3.1 Transcriptome‐wide analyses

Two‐way PERMANOVA (Table 1) identified strong and significant differences in transcriptome‐wide gene expression patterns between the three tissue types explaining 77% of the variation (*F* = 39.78, *r* ^2^ = 0.77, *df* = 2, *p* = .0001), but nonsignificant effects of pathogenic treatment (saline, heat‐killed, or live *S. marcescens*) (*F* = 0.86, *r* ^2^ = 0.02, *df* = 2, *p* = .47) or any of their interactions (*F* = 0.94, *r* ^2^ = 0.04, *df* = 4, *p* = .50). Because of these strong differences between tissue types, we ran three independent PERMANOVAs for each tissue type—maternal fat bodies and ovarioles as well as embryos—focused on identifying differences in gene expression due to pathogenic treatment.

**TABLE 1:** PERMANOVA of transcriptome‐wide expression for all tissue types

Factor	*df*	Sums of sqs	Mean sqs	F. Model	*R* ^2^	Pr(>*F*)
Tissue	2	0.865	0.432	39.778	0.772	0.0001
Treatment	2	0.019	0.009	0.863	0.016	0.472
Tissue:Treatment	4	0.041	0.010	0.938	0.036	0.498
Residuals	18	0.196	0.011		0.175	
Total	26	1.120			1.000	

**FIGURE 1:**
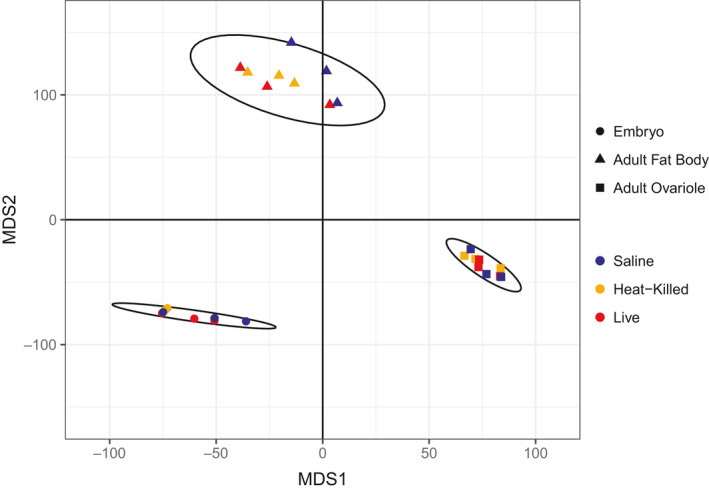
MDS of all tissue types showing clustering across tissue type (Embryo, Adult Fat Body, and Adult Ovariole) according to whether the mothers were exposed to heat‐killed *Serratia* (Heat‐Killed), live *Serratia* (Live), or saline (Saline) as a control

**FIGURE 2:**
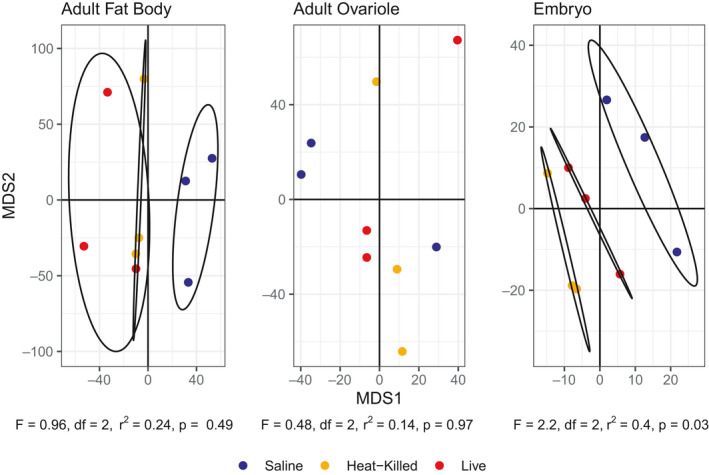
MDS of maternal fat body, maternal ovariole, and embryos showing clustering by maternal treatment to saline (Control), heat‐killed *Serratia* (Heat‐Killed), and live *Serratia* (Live). *F*, *r* ^2^, and *p*‐values are from PERMANOVA results of each tissue type

Although the independent PERMANOVAs did not identify significant differences in transcriptome‐wide gene expression due to maternal immune treatment in maternal fat bodies (*F* = 0.96, *r* ^2^ = 0.24, *df* = 2, *p* = .49) and ovarioles (*F* = 0.48, *r* ^2^ = 0.14, *df* = 2, *p* = .97), transcriptome‐wide patterns of gene expression in the embryos of the exposed mothers differed significantly (*F* = 2.2, *r* ^2^ = 0.44, *df* = 2, *p* = .03). In embryos, maternal immune treatment explained 44% of the variation in transcriptome‐wide gene expression. An MDS plot of all tissue types and treatments (Figure 1) also shows tissue type differences obscuring treatment differences. Separate MDS plots for each tissue type (Figure 2) show treatment differences within tissue types, with heat‐killed and live treatment groups clustering separately from the saline control for fat body and embryo but not ovariole.

### 3.2 **Gene‐**level analyses

Negative binomial GLMs were run separately for the two maternal tissues (fat body and ovarioles) as well as the embryos in order to identify genes that were differentially expressed due to heat‐killed or live *Serratia* exposure compared to the saline control (Table 2). Relative to the maternal saline treatment, fat bodies of heat‐killed *Serratia*‐treated mothers had 17 genes that were differentially expressed (DE). The fat bodies of live *Serratia*‐treated mothers had 99 genes that were DE. For the ovariole tissue, only one gene was differentially expressed (DE) for heat‐killed *Serratia‐*treated mothers. For the live *Serratia*‐injected mothers, their ovarioles also had one DE gene. In sharp contrast, embryos from heat‐killed *Serratia‐*treated mothers had 469 DE genes while embryos from live *Serratia‐*treated mothers had 150 DE genes.

**TABLE 2:** DE gene summary—all DE, annotated DE, and annotated DE with absolute LFC greater than 2 each tissue type (embryo, adult fat body, and adult ovariole) according to whether samples were treated with live or heat‐killed *Serratia* compared to the saline control

Tissue	Differentially expressed		Annotated		Annotated LFC > 2	
	Heat‐killed	Live	Heat‐killed	Live	Heat‐killed	Live
Embryo	469	150	334	106	31	15
Adult—Fat Body	17	99	7	57	5	51
Adult—Ovariole	1	1	0	1	0	1

**FIGURE 3:**
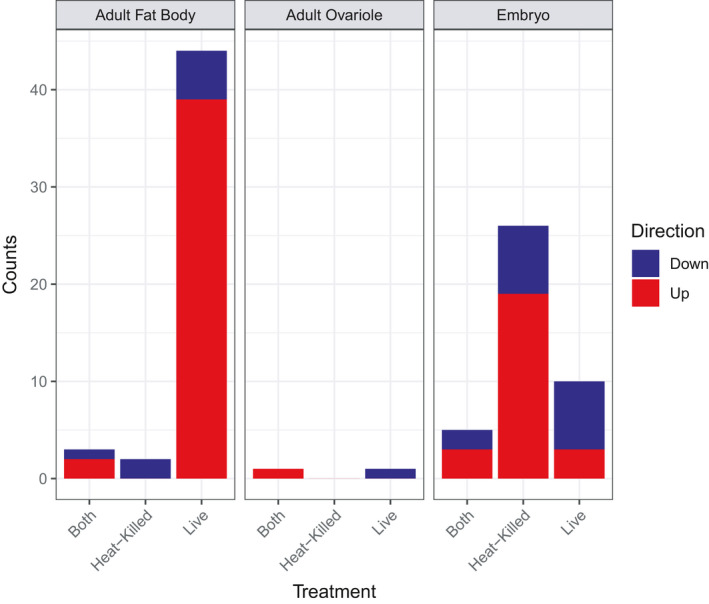
Differentially expressed genes by tissue (fat body, ovariole, nd embryo), treatment group (saline, heat‐killed, and live), and direction (upregulated or downregulated). Fat body had the greatest number of differentially expressed genes, the largest number being upregulated in response to live *Serratia* exposure. Second to fat body in terms of number of differentially expressed genes was embryo, the largest number being upregulated in response to maternal treatment with heat‐killed *Serratia*. Few genes were differentially expressed for ovariole

When KEGG gene annotations were identified for the DE genes, fat body had seven DE genes with annotations for heat‐killed exposures and 57 DE annotated genes for live exposures. Ovariole had zero DE annotated genes for heat‐killed exposure and one DE annotated gene for live exposures. Embryo had 334 DE annotated genes for heat‐killed and 106 live DE annotated genes. To focus on annotated genes showing the strongest effect, we focused on highly differentially expressed genes with absolute log_2_ fold change values greater than 2 (i.e., a five‐fold difference in gene expression). Among the highly differentially expressed genes, fat body had five heat‐killed and 51 live genes, ovariole had zero heat‐killed and one live gene, and embryo had 31 heat‐killed and 15 live genes (Table 2 and Figure 3).

**FIGURE 4:**
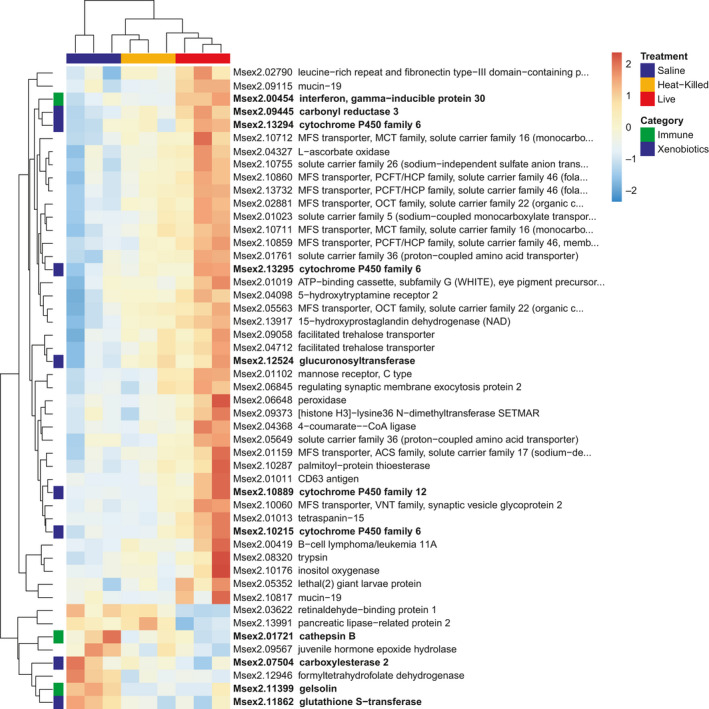
Heatmap normalized gene expression of differentially expressed (DE) genes in the adult fat body with a log_2_‐fold change (LFC) greater than 2 or less than −2. Exposure treatments (saline, live *Serratia*, and heat‐killed *Serratia*) color coded across top of heatmap. Hierarchical clustering of genes shown along vertical axis with immune genes and xenobiotic genes coded in green and blue, respectively

A heat map plotting highly DE genes for maternal fat body (Figure 4) shows that mothers treated with live *S. marcescens* strongly upregulated DE genes (41/49) relative to the more intermediate expression in the heat‐killed treatment and lower expression in the saline control. The remaining eight highly DE genes in maternal fat body were downregulated in the live treatment relative to the heat‐killed or saline treatments. Three of the highly DE genes were immune genes; interferon gamma‐inducible protein 30 was upregulated in live‐treated fat bodies, whereas cathepsin B and gelsolin were both downregulated. Eight of the highly DE genes were xenobiotic genes; six of which were upregulated in live‐treated fat bodies (carbonyl reductase 3, cytochrome P450 family 6 [Msex2.13294, Msex2.13295, Msex2.10215], glucuronosyltransferase, cytochrome P450 family 12); and two of which were downregulated (carboxylesterase 2, glutathione S‐transferase).

**FIGURE 5:**
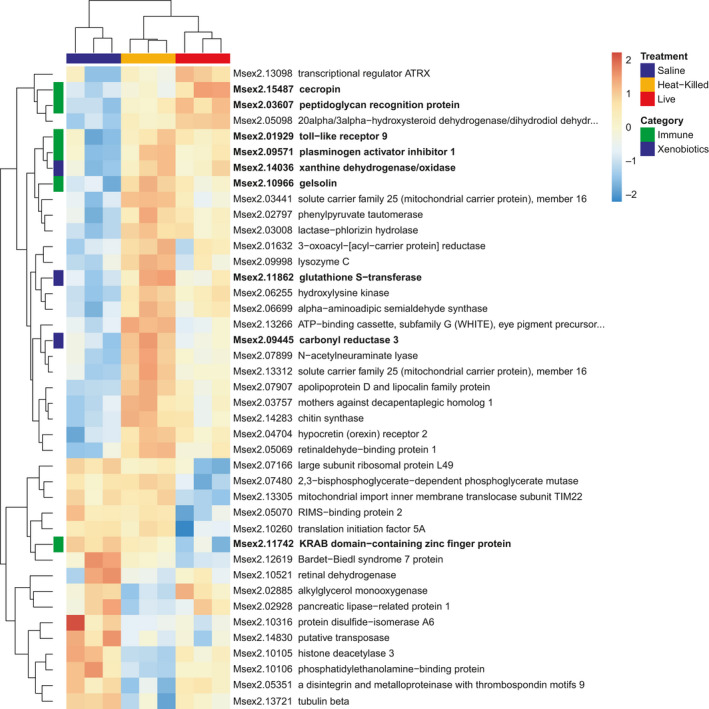
Heatmap normalized gene expression of differentially expressed (DE) genes in the embryos with a log_2_‐fold change (LFC) greater than 2 or less than −2. Exposure treatments (saline, live *Serratia*, and heat‐killed *Serratia*) color coded across top of heatmap. Hierarchical clustering of genes shown along vertical axis with immune genes and xenobiotic genes coded in green and blue, respectively

A heat map of highly DE genes for the embryos (Figure 5) showed clear clustering of the three treatment groups (saline, heat‐killed and live) with the heat‐killed and live treatments clustering together. Among the 25 highly DE genes that were upregulated in the heat‐killed and live exposure treatments, heat‐killed treatment generally showed higher expression than the live treatment. These 25 highly DE genes include five immune genes (cecropin, peptidoglycan recognition protein, toll‐like receptor 9, plasminogen activator inhibitor 1, gelsolin)—and three xenobiotic genes (xanthine dehydrogenase/oxidase, glutathione S‐transferase, carbonyl reductase 3) showing transgenerational effects. Sixteen highly DE genes were downregulated in the heat‐killed and live exposure treatments; nine DE genes were more downregulated in the heat‐killed treatment; and seven DE genes were more downregulated in the live treatment. Only one of the 16 downregulated genes (KRAB domain‐containing zinc finger protein) was an immune gene.

## 4 DISCUSSION

Our results showed a strong upregulation of genes in the fat bodies, but not ovarioles of adult female moths in response to injections of heat‐killed and live *Serratia*. Moreover, we observed a stronger upregulation of immune‐related genes in embryos from heat‐killed *Serratia*‐injected mothers than that from embryos exposed to live bacteria. This may be a result of mothers dealing with an active (i.e., live) *Serratia* infection engaging in trade‐offs and prioritizing their own survival over the transgenerational immune priming (TGIP) of their offspring, or possibly, due to the known immunosuppressive effect of *Serratia* (Ishii, Adachi, Hamamoto, & Sekimizu, 2014) which could have precluded TGIP if the mother's immune system was compromised by the immunosuppressive compounds synthesized by *Serratia*. Higher numbers of DE immune genes in the offspring of mothers treated with heat‐killed *Serratia*, on the other hand, provide strong evidence for TGIP. Interestingly, the DE genes observed in the immune responses of adult moths versus via transgenerational priming do not mirror each other. The DE immune‐related genes differed between mothers and their offspring, with xenobiotics predominating in the mother's response, and PRRs and antimicrobial peptides predominating in the TGIP response.

Patterns of differential expression of genes can be characterized as sets of DE immune and xenobiotic genes that were (a) similarly upregulated for both adults and embryos, (b) DE of immune and xenobiotic genes only in mothers (but not embryos), or (c) DE of immune and xenobiotic genes only in embryos of treated mothers. We discuss these groups of genes below. A complete list of differentially expressed genes, annotations, annotation identities, and e‐values is available in Appendix S1.

### 4.1 Shared upregulated adult and offspring xenobiotic genes

There was very little overlap of shared DE xenobiotic genes detected in the adults and transgenerationally primed embryos. Only carbonyl reductase 3 (CBR3/Msex2.09445) was upregulated in adults treated with live *Serratia* and embryos whose mothers were exposed to heat‐killed *Serratia*. Upregulation of carbonyl reductases has been reported in gypsy moth (*Lymantria dispar*) on protein‐deficient diets (Lindroth, Barman, & Weisbrod, 1991). Upregulation of carbonyl reductase 3 has also been reported in human cancer cells in response to oxidative stress (Ebert, Kisiela, Malátková, El‐Hawari, & Maser, 2010). To the best of our knowledge, this is the first report of upregulation in response to immune challenge in Lepidoptera.

### 4.2 Upregulated adult‐only immune and xenobiotic genes

The adult‐only response was characterized by strong upregulation of xenobiotic genes in mothers injected with live bacteria. Cytochrome P450 family 12 (CYP12/Msex2.10889) and cytochrome P450 family 6 (CYP6/Msex2.13294, CYP6/Msex2.10215, and CYP6/Msex2.13295) were upregulated in adults exposed to live *Serratia*. CYP12 and CYP6 are members of the cytochrome P450 family involved in oxidation/reduction of organic chemicals including drugs, environmental toxins, and carcinogens in humans (Guengerich, Waterman, & Egli, 2016). Upregulation of cytochrome P450 has been reported in several invertebrates including silkworms (*Bombyx mori*) challenged with *B. thuringiensis* (Wu & Yi, 2018), flour beetles (*T. castaneum*) challenged with LPS (Altincicek et al., 2013), and abalone (*Haliotis diversicolor*) challenged with gram‐negative and gram‐positive bacteria (Wang, Ren, Xu, Cai, & Yang, 2008). The role upregulation of cytochrome P450 plays in the insect immune response is unclear, but Wu and Yi (2018) proposed that this may be a detoxification response to protect the host from intermediate metabolites involved in the energetic cost of mounting an immune response. Glucuronosyltransferase (UGT/Msex2.12524) was also upregulated when mothers were injected with live *Serratia*. UGT catalyzes the attachment of sugars to toxins to facilitate their excretion (Meech, Miners, Lewis, & Mackenzie, 2012). Upregulation of UGT in response to immune challenge has been reported in *Drosophila* exposed to *E. coli* (Johansson, Metzendorf, & Söderhäll, 2005), and perhaps its upregulation helps *Manduca* mothers detoxify the toxins produced by *Serratia*.

Only one immune gene was strongly upregulated in mothers injected with live bacteria—interferon gamma‐inducible protein 30 (GILT). GILT is involved in MHC antigen processing in mammals (Jensen, 1993), and upregulation of GILT in response to immune challenge has been identified in several invertebrates including disk abalone exposed to gram‐negative *Vibrio alginolysticus* (Zoysa & Lee, 2007), mosquito exposed to the malarial parasite *Plasmodium falciparum* (Schleicher et al., 2018), and fruit fly exposed to gram‐negative *E. coli* (Kongton et al., 2014).

### 4.3 Downregulated adult‐only immune and xenobiotic genes

Carboxylesterase 2 (CES2/Msex2.07504) and cathepsin B (CTSB/Msex2.01721) were downregulated within fat body in mothers exposed to live *Serratia*. It is unclear why these two genes were downregulated as we find no comparative studies showing a similar pattern. CES2 catalyzes the metabolism of ester and pyrethroid toxins (Wang et al., 2018). Cathepsins are proteases expressed in lysosomes. CTSB is highly expressed in the fat body of *B. mori* during the larval–pupal transformation, and it is involved in the programmed cell death of the fat body during metamorphosis (Lee et al., 2009). In addition to its role in metamorphosis, cathepsin B has been associated with the response to immune challenge. Wu et al. (2011) reported upregulation of cathepsins B and D in *B. mori* challenged with *B. mori* nuclear polyhedrosis virus (BmNPV). Our results showed a counter‐intuitive pattern in *M. sexta* (downregulation) relative to results reported by Wu et al. (2011) in the closely related species *B. mori*, but this downregulation could be a host response to the known immunosuppressive effects of *S. marcescens* (Ishii et al., 2014).

### 4.4 Signatures of TGIP in Embryos

Compared to the maternal response, the transgenerational immune responses in embryos of mothers who experienced heat‐killed *Serratia* injections (Figure 5) involved strong upregulation of immune genes—cecropin (CEC), peptidoglycan recognition protein (PGRP), toll‐like receptor 9 (TLR9), and plasminogen activator inhibitor 1 (PAI1).

Cecropins are antimicrobial peptides active against many gram‐negative bacteria and fungi (Lee et al., 2013). Contreras‐Garduño et al. (2015) reported within‐generation immune priming and upregulation of cecropin in marsh mosquitoes (*Anopheles albimanus*) exposed to the protozoan parasite *Plasmodium berghei*, but to our knowledge, we are the first to document transgenerational upregulation of cecropin in an insect.

PGRP is involved in the response to gram‐positive bacteria via the toll pathway and gram‐negative bacteria like *Serratia* via the IMD pathway (Gottar et al., 2002). Freitak et al. (2014) reported transgenerational upregulation of PGRP in the eggs of immune‐challenged *M. sexta* mothers, and Eggert et al. (2014) reported upregulation PGRP in the offspring of *T. castaneum* fathers exposed to *B. thuringiensis*.

Msex2.01919, whose best BLAST hit was for mammalian TLR9, was upregulated in embryos of mothers exposed to heat‐killed *Serratia*. In mammals, toll‐like receptors are primarily involved in immune responses, while in insects, toll‐like receptors contribute to both development and immune response roles (Imler & Zheng, 2004). In mammals, TLR9 binds CpG motifs in bacterial DNA (Cornelie et al., 2004). Upregulation of other toll‐like homologues (TLR4 homolog MsToll) has been reported in *M. sexta* challenged with gram‐negative bacteria (*E. coli*), gram‐positive bacteria (*M. lysodeikticus*), and yeast (*Saccharomyces cerevisiae*) (Ao et al., 2008).

Plasminogen activator inhibitor 1 (PAI‐1) was upregulated in embryos of mothers exposed to heat‐killed *Serratia*. PAI‐1 is a serine protease inhibitor involved in hemostasis in mammals (Lijnen, 2005). PAI‐I is upregulated in humans during gram‐negative sepsis caused by *Burkholderia pseudomallei* and protects the host by limiting bacterial growth, inflammation, and coagulation (Kager et al., 2011). We believe we are the first to report transgenerational PAI‐1 upregulation in invertebrates, and it is possible that PAI‐1 plays a similar protective anticoagulation role in *Manduca*.

## 5 CONCLUSION

Maternal exposure of *M. sexta* to both heat‐killed and live *S. marcescens* had strong and significant transgenerational impacts on gene expression patterns of their offspring, and these patterns include upregulation of genes that could play a role in the transgenerational phenotypic effects reported by Rosengaus et al. (2017). This combination of phenotypic and transcriptomic transgenerational effects adds to the growing body of evidence for transgenerational immune priming in insects. Our results indicate that immune priming includes upregulation of genes associated with pathogen recognition, pathogen elimination, and modulation of downstream effects like coagulation. Further exploration is warranted to determine the mechanisms that drive these TGIP gene expression changes.

## CONFLICT OF INTEREST

None declared.

## AUTHOR CONTRIBUTIONS

**Charles L. Roesel:** Data curation (lead); formal analysis (lead); methodology (equal); software (lead); validation (equal); visualization (lead); writing – original draft (lead); writing – review and editing (equal). **Rebeca B. Rosengaus:** Conceptualization (lead); formal analysis (equal); funding acquisition (equal); investigation (equal); methodology (lead); project administration (equal); supervision (equal); validation (equal); writing – review and editing (equal). **Wendy Smith:** Conceptualization (lead); formal analysis (equal); funding acquisition (equal); investigation (equal); methodology (equal); project administration (equal); supervision (equal); validation (equal); writing – review and editing (equal). **Steven V. Vollmer:** Conceptualization (lead); formal analysis (equal); funding acquisition (equal); investigation (equal); methodology (equal); project administration (equal); supervision (lead); validation (equal); writing – review and editing (equal).

## Supporting information

Appendix S1

## Data Availability

The Illumina RNA‐Seq read data are available on NCBI SRA https://www.ncbi.nlm.nih.gov/sra under BioProject accession number PRJNA603121. Normalized read count data, DESeq2, and annotation files (.csv) are available on Dryad https://doi.org/10.5061/dryad.wpzgmsbjt.
